# Seroprevalence and associated risk factors of peste des petits ruminants in sheep and goats in three districts of the Central Oromia Region, Ethiopia

**DOI:** 10.3389/fvets.2024.1402342

**Published:** 2024-11-13

**Authors:** Tilahun Guday Wendimu, Tegegn Dilbato Dinbiso, Demeke Sibhatu Lobago, Lencho Megersa Marami

**Affiliations:** ^1^Livestock Health Core Work Process, Dano District Agriculture Office, Dano, Oromia, Ethiopia; ^2^Department of Veterinary Science, School of Veterinary Medicine, Ambo University, Ambo, Oromia, Ethiopia; ^3^Department of Viral Serology, Animal Health Institute, Sebeta, Oromia, Ethiopia; ^4^Department of Veterinary Laboratory Technology, School of Veterinary Medicine, Ambo University, Ambo, Oromia, Ethiopia

**Keywords:** Ethiopia, goat, peste des petits ruminants, risk factors, seroprevalence, sheep

## Abstract

**Background:**

Peste des petits ruminants (PPR) is a viral disease that affects domestic and wild small ruminants and camels in Africa, the Middle East, and Asia. Following the successful eradication of rinderpest, the World Organization for Animal Health (WOAH) and the Food and Agriculture Organization (FAO) have undertaken to eradicate PPR by 2030. Regular surveillance and monitoring of the disease in various regions of Ethiopia are crucial to achieving this goal. This study aimed to estimate the prevalence of PPR, assess community awareness of PPR infection, and examine the associated risk factors of the disease in selected districts of the Central Oromia Region, Ethiopia.

**Method:**

The study collected 384 serum samples from 73 flocks containing 217 sheep and 167 goats using a multi-stage sampling technique. A competitive enzyme-linked immunosorbent assay (c-ELISA) was used to detect antibodies against the PPR virus. Additionally, a pre-tested questionnaire was used to gather information on community awareness and potential risk factors for PPRV infection in the study area.

**Results:**

The study found that the overall prevalence of PPR in flocks was 71.2% [95% confidence interval (CI): 59.4%−81.2%]. The prevalence of PPR at the animal level was 50% (95% CI: 44.9%−55.1%), with sheep having a prevalence of 54.4% (95% CI: 47.0%−60.6%) and goats having a prevalence of 44.3% (95% CI: 36.6%−52.2%). The study also found that districts, flock size, and agroecology were independent predictors of PPRV seropositivity in sheep, whereas districts, origin, and mixed species were independent predictors of PPRV seropositivity in goats.

**Conclusion:**

The study revealed a high prevalence of PPR in sheep and goats in the study area. To prevent the spread of the disease, the study suggests quarantining animals before introducing them to districts, regular PPR vaccination, and isolation and molecular characterization of the PPR virus circulating in the study area.

## 1 Introduction

Peste des petits ruminants (PPR), also known as goat plague, is the most economically important viral disease of sheep, goats, and some wild small ruminants and camels (1, 2). It is a contagious transboundary disease first reported in Côte d'Ivoire in 1942 (3). The morbidity and mortality rates of the disease in susceptible populations can reach 90%−100% and 50%−100%, respectively (4). The annual loss in PPR-related costs is estimated to be $1.4–2.1 billion globally. This disease affects nearly 30 million animals, mainly goats, and sheep, annually across over 70 countries worldwide (1).

Peste des petits ruminants (PPR) is caused by the peste des petits ruminants virus (PPRV) of the genus *Morbillivirus* (5, 6). The ICTV has renamed the species of PPRV from *Small ruminant morbillivirus* to *Morbillivirus caprinae* (6–8). PPR virus has a single serotype but four different genetic lineages (9). PPR is an acute disease in goats and sheep, and the incubation lasts for 3–4 days. It produces pyrexia followed by the onset of other clinical signs, including watery oculo-nasal discharge, congestion of the mucous membranes of the buccal cavity, conjunctiva of the eye, and the vulva (10, 11). An immunocapture ELISA or polymerase chain reaction is used for PPR diagnosis. In addition, counter immunoelectrophoresis and agar gel immunodiffusion may also be used. Virus neutralization tests and competitive ELISA have been used as serological tests (12).

The PPR case was diagnosed and reported for the first time in 1941 in Côte d'Ivoire, then after three decades the disease's clinical signs were identified in goat flocks in the Afar Region of Ethiopia (13). The virus was officially confirmed in 1991 near Addis Ababa using cDNA probes from lymph nodes and spleen samples collected during an outbreak of PPR in goat markets (14). In 2014, the complete PPRV genome was sequenced for the first time in Ethiopia from infected goats during an outbreak and was identified as belonging to lineage IV (15). Recently, six complete PPRV genomes of Ethiopian origin have been sequenced and collected in GenBank (16, 17).

Recently, various researchers from Ethiopia reported an overall seroprevalence of PPR in sheep and goats in many regions of the country, such as 60.15% in Afar, 18.3% in the Amhara Region, 12.9% in Bale Zone, Oromia Region, and 29.2 in Gurage Zone, South Region (18–21).

Among PPR-endemic countries, Ethiopia ranks seventh in the small livestock population, which accounts for the largest share of national meat consumption and export earnings (22, 23). The small ruminant population of Ethiopia is about 39,894,394 sheep and 50,501,672 goats (24). Since the first cases of PPR in 1977 in Ethiopia, the disease has continued to affect smallholder livestock production and food security. Thus, the WOAH and the FAO created the Global Control and Eradication Strategy to eradicate PPR by 2030. Ethiopia plans to eradicate the disease by 2027 (25).

Districts in the Oromia Regional State that share a border with the Afar Region are at a high risk of PPR virus infection due to frequent and uncontrolled livestock movements along the border (16). The small ruminant population in the area changes rapidly, resulting in an increase in unvaccinated small ruminants, which contributes to the occurrence and maintenance of the disease (26). Accurate and timely data on the distribution of PPR infection and the assessment of animal and herd level risk factors are critical in controlling the disease and achieving eradication. However, despite its importance, the epidemiological risk factors and linkages between continuing outbreaks and the spread of PPR are not well understood, necessitating further studies. The aim of the current study is to estimate the PPR seroprevalence and assess associated risk factors in selected districts of the Central Oromia Regional State, Ethiopia.

## 2 Materials and methods

### 2.1 Study area description

**Figure 1:**
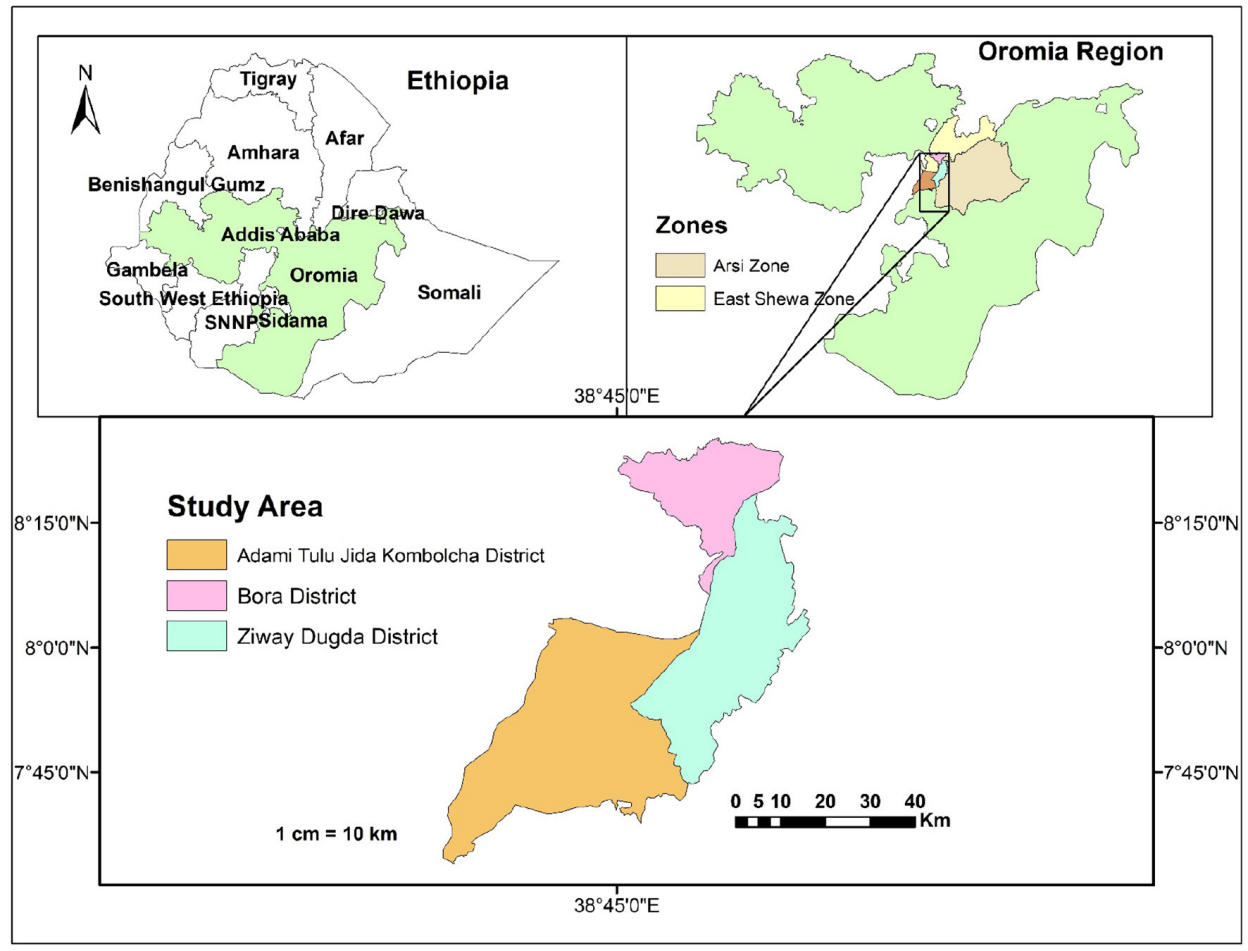
Map of the study area.

The study was conducted in Adami Tullu Jido Kombolcha (ATJK), Bora, and Ziway Dugda districts of the Central Oromia Region (Figure 1).

The ATJK district is in the Great Rift Valley. Adami Tullu is the principal town in the district. Most of this district ranges in altitude from 1,500 to 2,300 m above sea level (m.a.s.l). ATJK has a latitude of 7^o^52′0″ North and a longitude of 38^o^42′0″ East with an elevation of 1,643 m.a.s.l. The rivers found in the districts are Bulbula, Jido, Hora Kalio, and Gogessa. This district has an estimated total population of 167,066, of whom 82,926 are men and 84,140 are women; 57,068 or 34.16% of its population are urban dwellers (27). The district has 418,301 estimated livestock population, including 163,164 cattle, 43,050 sheep, 83,874 goats, 31,836 donkeys, 4,034 mules, 22,231 horses, and 70,112 poultry.

The Bora district is also in the Great Rift Valley. The total human population of the district is 58,748, of whom 30,487 are men and 28,261 are women; 11,403, or 19.41% of them, are urban dwellers (27). The total number of livestock is 774,456, including 87,715 cattle, 48,449 sheep, 39,212 goats, 6,735 donkeys, 809 mules, 1,224 horses, and 590,312 poultry.

The Ziway Dugda district is in the Great Rift Valley. The altitude of this district ranges from 1,500 to 2,300 m.a.s.l. It is geographically situated between latitude: 8°00′0.00″N and longitude: 39°00′0.00″E. This district has an estimated population of 120,121, of whom 60,700 are men and 59,421 are women; 4,338 or 3.61% of them are urban dwellers (27). The total livestock in the district is 300,849, including 119,072 cattle, 53,815 sheep, 31,102 goats, 10,644 donkeys, 1,178 mules, 24,175 horses, and 60,863 poultry.

### 2.2 Study population

The study population comprised all local sheep and goat breeds that had not received the PPR vaccine and were at least 6 months old. The PPR vaccination status of study animals were confirmed using district documents, field veterinarians, and community animal health workers. Small ruminants that had had vaccinations were not included in the study because the c-ELISA used in this investigation could not distinguish between antibodies produced by vaccination and those produced from a natural illness. Because of maternal antibodies, only sheep and goats older than 6 months were allowed to take part in the study (28).

### 2.3 Study animal

The determination of age of sheep and goats was according dental pattern (dentition) (29, 30) and grouped into young (6–18 months) and adults (>18 months). The number of sheep and goats per flock was categorized into small (< 30), medium (29–49), and large (>50).

### 2.4 Study design

A cross-sectional study design was carried out from November 2019 to May 2021 in the selected district of the Central Oromia Region, Ethiopia. In addition, a semi-structured questionnaire survey was used to assess risk factors and community awareness regarding PPRV infection.

### 2.5 Sample size determination and sampling methods

A sample size of study animals was calculated using the formula described by Thrusfield (31) which is $n\;=\;\frac{Z^{2}\;P_{\exp}\left(1-P_{\exp}\right)}{d^{2}}$. Where *Z* = 1.96, *n* = sample size, *P*_exp_ = expected prevalence, and *d* = absolute precision.

Gari (32) reported an overall PPR seroprevalence of 48.43% from the Arsi Zone. Based on this data, the sample size for the current study was calculated using the estimated prevalence of 48.43, the 95% confidence interval, and the desired absolute precision of 5%. So, the sample size for small ruminants was 384.

A multi-stage sampling method was used. First, three districts were chosen randomly. Then, seven peasant associations that include three villages were selected from each district. Out of 21 villages considered, 73 flocks of small ruminants, including 217 sheep and 167 goats, were randomly selected.

For a questionnaire survey, the sample size was calculated using the formula given by Arsham (33) which is as follows: $N\;=\;\frac{0.25}{SE^{2}}$, Where *N* = sample size and SE (standard error) = 5%. Thus, the calculated sample size was 100.

### 2.6 Sample collection and transportation

In this study, 384 sera samples were collected from 217 sheep and 167 goats. Initially, a blood sample was collected from the jugular vein of each animal using a plain vacutainer tube and labeled. Then, the tube that had the blood sample was kept in a slant position overnight to allow for serum separation from the clotted blood. Then the serum was decanted and aliquoted into 1.5 ml cryovials, labeled, and kept in an icebox. Finally, the sera samples were shipped in a cool box chilled on ice packs to the Animal Health Institute at Sebeta and stored at 20°C until serological testing.

### 2.7 Serological investigation

A competitive enzyme-linked immunosorbent assay (c-ELISA) (IDVet Innovative Diagnostics, France) was used to detect antibodies against PPRV in sera samples (34). According to the manufacturer, the specificity and sensitivity of c-ELISA at animal and flock levels are 99.4 and 94.5%, respectively. Briefly, the ELISA plates were coated with a PPR antigen. The unbound antigen was washed away using a buffer, and a serum sample was added. A rabbit anti-mouse-antibody horseradish peroxidase (HRPO) conjugate was added and incubated with constant agitation at each stage. A substrate solution (O-phenylenediamine dihydrochloride having H_2_O_2_) was added, allowing a color reaction to develop, halted with an equal volume of 1 M H_2_SO_4_. The optical density value was recorded at 450 nm using the ELISA plate reader according to the manufacturer's instructions. The apparent prevalence was used to report the result of the present study. The seroprevalence of PPR at the flock level was considered if a single animal or more animals tested positive by a serological test.

### 2.8 Data collection

All required epidemiological information on individual animals and flock levels that needed to be considered for PPR risk factors was obtained from the animal owner in addition to the collection of blood samples. Hypothesized risk factors considered were the sex of animals (female, male), age [young (6–18 months), adult (>18 months)], species (sheep, goats), flock size [small (< 30), medium (29–49), large (>50)], mixed species (yes, no), the introduction of a new animal to a flock (yes or no), the origin of animal (born, brought), grazing (zero grazing, fenced, communal), water source (at a farm, shared), districts (Ziway Dugda, Bora, ATJK) and agroecology [lowland (< 1,500 masl), midland (1,500–2,300 masl), and highland (>2,300 masl)].

#### 2.8.1 Questionnaire survey

A pre-tested questionnaire survey was developed to gather data about the socio-demographic characteristics, PPR knowledge, and animal husbandry practices of respondents. The questionnaire was prepared in English and translated into the local language “Afan Oromo.” The survey consisted of closed-ended questions with binary and multiple-choice options. Participants were animal owners who were at least 15 years old, living in peasant associations, and able to communicate verbally in the local language. The selection of participants for the survey was random. Information on the socio-demographic profiles, production systems, reasons for keeping small ruminants, market information, symptoms, seasons of outbreak occurrence, and PPR vaccination status were collected from the respondents.

### 2.9 Data analysis

Data gathered from serological and questionnaire surveys were entered into a Microsoft Excel spreadsheet (Microsoft Corporation, 2016) and analyzed using Stata 14. The potential risk factors considered in the data analysis were age, species, herd size, mixed species, the introduction of a new animal to a flock, the origin of the animal, grazing, water source, districts, and agroecology. The association between risk factors and seroprevalence of PPR at the flock level was assessed using Pearson's chi-squared test. Using univariable and multivariable logistic regression, the crude odds ratio and adjusted odds ratio at a 95% CI were calculated, respectively. The Variance Inflation Factor (VIF) was used to detect multicollinearity. Those non-collinear variables that had a *p*-value of < 0.25 during univariable logistic regression analysis were considered for multivariable logistic regression. A binomial exact test was used to calculate the 95% CI of the prevalence estimates. A statistically significant difference was considered when the *p*-value was < 0.05.

### 2.10 Ethical clearance

The proposal was critically reviewed for a potential ethical concern by the Guder Mamo Mezemer Campus Ethical Review Committee of Ambo University, and it was approved. First, the study's purpose and description were explained to participants and animal owners, and these volunteers agreed to take part. The authors used code to handle the data gathered to ensure the confidentiality of personnel data. Animals were handled with the best veterinary care, and blood samples were collected according to standard protocols.

## 3 Results

### 3.1 Overall seroprevalence

Out of the 73 flocks of sheep and goats tested, 52 (71.2%, 95 CI: 59.4–81.2) were found to be PPR seropositive. A higher PPR seroprevalence at the individual animal level was recorded in the Bora district (76.2% CI: 67.8–83.3), followed by the ATJK district (62.1% CI: 49.3–73.8) and the Ziway Dugda district (28.7% CI: 22.4–35.6). There was a significant relationship between districts and PPRV seropositivity at the individual (χ^2^ = 73.4710, *p*-value < 0.001) and flock level (χ^2^ = 21.1131, *p*-value < 0.001; Table 1).

**Table 1:** Seroprevalence of PPR in sheep and goats at the individual animal and flock levels.

**Districts**	**Animal level**			**Flock level**		
	**No. tested**	**No. positive (%)**	**95% CI**	**No. tested**	**No. positive (%)**	**95% CI**
ATJK	66	41 (62.1)	49.3–73.8	9	8 (88.9)	51.8–99.7
Bora	126	96 (76.2)	67.8–83.3	25	25 (100.0)	86.3–100
Ziway Dugda	192	55 (28.7)	22.4–35.6	39	19 (48.7)	32.4–65.2
Total	384	192 (49)	44.9–55.1	73	52 (71.2)	59.4–81.2
Chi-squared test	χ^2^ = 73.4710 *p*-value < 0.001^*^			χ^2^ = 21.1131 *p*-value < 0.001^*^		

χ^2^, chi-square.

^*^Statistically significant.

### 3.2 Risk factors of PPR at flock level

The seroprevalence of PPR at the flock level in sheep was almost equal to that in goats. A higher seroprevalence of PPR was recorded in the lowland than in the highland. Potential risk factors that were statistically significantly associated with the seroprevalence of PPR at the flock level include district (χ^2^ = 21.1131, *p*-value < 0.001), agroecology (χ^2^ = 13.1342, *p*-value = 0.001), and the introduction of a new animal into a flock (χ^2^ = 6.1406, *p*-value = 0.013; Table 2).

**Table 2:** Association between risk factors and PPRV seropositivity in sheep and goats flocks using Chi-square test.

**Variables**	**Categories**	**No. tested**	**No. positive**	**Prevalence (95% CI)**	**χ^2^**	***p*-value**
Districts	ATJK	9	8	88.9 (51.8–99.7)	21.1131	< 0.001^*^
	Bora	25	25	100.0 (86.3–100.0)		
	Ziway Dugda	39	19	48.7 (32.4–65.2)		
Species	Sheep	40	28	70.0 (53.5–83.4)	0.0656	0.798
	Goats	33	24	72.7 (54.5–86.7)		
Flock size	Small	49	32	65.3 (50.4–78.3)	5.8082	0.055
	Medium	12	8	66.7 (34.9–90.0)		
	Large	12	12	100 (73.5–100.0)		
Agroecology	Highland	20	8	40.0 (19.1–64.0)	13.1342	0.001^*^
	Midland	34	28	82.4 (65.5–93.2)		
	Lowland	19	16	84.2 (60.4–96.6)		
Introduction of a new animal	No	39	23	59.0 (42.0–74.4)	6.1406	0.013^*^
	Yes	34	29	85.3 (69.0–95.0)		
Mixed species	No	3	3	100.0 (29.2–100.0)	1.2635	0.261
	Yes	70	49	70.0 (57.9–80.4)		
Grazing	Communal	63	44	69.4 (57.0–80.8)	1.2712	0.530
	Fenced	7	5	71.4 (29.0–96.3)		
	Zero grazing	3	3	100.0 (29.2–100.0)		
Water source	Shared	63	44	69.8 (57.0–80.8)	0.4246	0.510
	At farm	10	8	80 (44.4–97.5)		

χ^2^, chi-square.

^*^Statistically significant.

### 3.3 Risk factors of PPR at the individual level

#### 3.3.1 Risk factors of PPR in sheep

Univariable logistic regression analysis showed that there was an association between PPR seropositivity in sheep and risk factors, including district [ATJK district (Crude odds ratio (COR): 6.0, 95% CI: 2.4–14.6, *p* < 0.001), Bora district (COR: 7.0, 95% CI: 3.7–13.4, *p* < 0.001)], agroecology [midland (COR: 8.8, 95% CI: 3.7–20.9, *p* < 0.001), lowland (COR: 9.1, 95% CI: 3.7–22.7, *p* < 0.001)] and flock size [medium (COR: 1.6, 95% CI: 0.8–3.2, *p* = 0.192), large (COR: 2.3, 95% CI: 1.2–4.8, *p* = 0.018)] which were statistically significant differences (*p* < 0.05; Table 3).

**Table 3:** Univariable logistic regression analysis of potential risk factors associated with Sheep PPR seroprevalence.

**Variables**	**Categories**	**No. of tested**	**No. of positive (prevalence)**	**OR (95% CI)**	***p*-value**
Districts	Ziway Dugda	93	27 (29.0)	1	
	ATJK	31	22 (71.0)	6.0 (2.4–14.6)	< 0.001^*^
	Bora	93	69 (74.2)	7.0 (3.7–13.4)	< 0.001^*^
Sex	Male	39	21 (53.9)	1	
	Female	178	97 (54.5)	1.0 (0.5–2.1)	0.941
Water source	Shared	201	108 (53.7)	1	
	on farm	16	10 (62.5)	1.4 (0.5–4.1)	0.500
Agroecology	Highland	47	8 (17.0)	1	
	Midland	104	67 (64.4)	8.8 (3.7–20.9)	< 0.001^*^
	Lowland	66	43 (65.2)	9.1 (3.7–22.7)	< 0.001^*^
Age	Young	36	16 (44.4)	1	
	Adult	181	102 (56.4)	1.6 (0.8–3.3)	0.193
Origin	Born	170	92 (54.1)	1	
	Brought	47	26 (55.3)	1.0 (0.5–2.0)	0.884
Flock size	Small	126	60 (47.6)	1	
	Medium	44	26 (59.1)	1.6 (0.8–3.2)	0.192
	Large	47	32 (68.1)	2.3 (1.2–4.8)	0.018^*^
Mixed species	Yes	206	110 (53.4)	1	
	No	11	8 (72.7)	2.3 (0.6–9.0)	0.222
Grazing	Fenced	8	3 (37.5)	1.0	
	Communal	201	108 (53.7)	1.9 (0.5–8.3)	0.375
	Zero Grazing	8	7 (87.5)	11.7 (0.9–147.6)	0.058

^*^Statistically significant.

#### 3.3.2 Risk factors of PPR in goats

As shown in Table 4, district [ATJK district (COR: 3.0, 95% CI: 1.4–6.7, *p* = 0.007), Bora district (COR: 11.4, 95% CI: 4.3–30.6, *p* < 0.001)], agroecology [midland (COR: 3.8, 95% CI: 1.7–8.6, *p* = 0.001), lowland (COR: 16.4, 95% CI: 4.9–54.4, *p* < 0.001)], flock size [medium (COR: 1.6, 95% CI: 0.6–4.1, *p* = 0.350), small (COR: 2.3, 95% CI: 1.1–4.5, *p* = 0.019)] and mixed species [yes (COR: 6.3, 95% CI: 2.3–17.1, *p* < 0.001)] were potential risk factors that had statistically significant associations with PPRV seropositivity in goats.

**Table 4:** Univariable logistic regression analysis of potential risk factors associated with goats' PPR seroprevalence.

**Variables**	**Categories**	**No. of tested**	**No. of positive (prevalence)**	**OR (95% CI)**	***p*-value**
Districts	Ziway Dugda	99	28 (28.3)	1	
	ATJK	35	19 (54.3)	3.0 (1.4–6.7)	0.007^*^
	Bora	33	27 (81.8)	11.4 (4.3–30.6)	
Sex	Male	30	10 (33.3)	1	
	Female	137	64 (46.7)	1.8 (0.8–4.0)	0.185
Water source	On farm	34	14 (41.2)	1	
	Shared	133	60 (45.1)	1.2 (0.5–2.5)	0.680
Agroecology	Highland	51	10 (19.6)	1	
	Midland	91	44 (48.4)	3.8 (1.7–8.6)	0.001^*^
	Lowland	25	20 (80.0)	16.4 (4.9–54.4)	< 0.001^*^
Age	Young	49	19 (38.8)	1	
	Adult	118	55 (46.6)	1.4 (0.7–2.7)	0.354
Origin	Born	145	61 (42.0)	1	
	Brought	22	13 (59.1)	2.0 (0.8–4.9)	0.139
Flock size	Large	63	21 (33.3)	1	
	Medium	25	11 (44.0)	1.6 (0.6–4.1)	0.350
	Small	79	42 (53.2)	2.3 (1.1–4.5)	0.019^*^
Mixed species	No	34	5 (14.7)	1	
	Yes	133	69 (51.9)	6.3 (2.3–17.1)	< 0.001^*^
Grazing	Fenced	8	3 (37.5)	1	
	Zero grazing	26	11 (42.3)	1.2 (0.2–6.2)	0.809
	Communal	133	60 (45.1)	1.4 (0.3–6.0)	0.675

^*^Statistically significant.

According to multivariable logistic regression analysis, districts [ATJK district (AOR: 3.6, 95% CI: 1.1–12.1, *p* = 0.036), Bora district (AOR: 9.7%, 95% CI: 3.7–25.5, *p* < 0.001)], flock size [medium (AOR: 3.4, 95% CI: 1.4–8.7, *p* = 0.009), large (AOR: 9.0, 95% CI: 3.2–25.2, *p* < 0.001)], agroecology, [midland (AOR: 2.1, 95% CI: 0.7–6.4, *p* = 0.215), lowland (AOR: 3.8, 95% CI: 1.2–11.7, *p* = 0.019)] were independent predictors of PPRV seropositivity in sheep, while districts [ATJK district (AOR: 3.0, 95% CI: 1.0–8.9, *p* = 0.043), Bora district (AOR: 9.7%, 95% CI: 2.9–32.8, *p* < 0.001)], origin [brought (OR: 4.5, 95% CI: 1.3–15.5)], mixed species [yes (OR: 7.2, 95% CI: 1.7–30.2, *p* = 0.007)] were independent predictors of PPRV seropositivity in goats (Table 5).

**Table 5:** Result of multivariable logistic regression analysis of potential risk factors of PPR seroprevalence in sheep and goats.

**Species**	**Variables**	**Category**	**OR (95% CI)**	***p*-value**
Sheep	Districts	Ziway Dugda	1	
		ATJK	3.6 (1.1–12.1)	0.036^*^
		Bora	9.7 (3.7–25.5)	< 0.001^*^
	Age	Young	1	
		Adult	1.7 (0.7–4.1)	0.244
	Flock Size	Small	1	
		Medium	3.4 (1.4–8.7)	0.009^*^
		Large	9.0 (3.2–25.2)	< 0.001^*^
	Mixed species	Yes	1	
		No	1.7 (0.4–7.3)	0.501
	Grazing	Fenced	1	
		Communal	0.6 (0.1–5.7)	0.679
		Zero grazing	1.6 (0.1–34.4)	0.752
	Agroecology	Highland		
		Midland	2.1 (0.7–6.4)	0.215
		Lowland	3.8 (1.2–11.7)	0.019^*^
Goats	Districts	Ziway Dugda	1	
		ATJK	3.0 (1.0–8.9)	0.043^*^
		Bora	9.7 (2.9–32.8)	< 0.001^*^
	Sex	Male	1	
		Female	1.2 (0.5–3.5)	0.670
	Origin	Born	1	
		Brought	4.5 (1.3–15.5)	0.017^*^
	Flock size	Large	1	
		Medium	0.3 (0.1–1.3)	0.104
		Small	0.4 (0.1–1.1)	0.079
	Mixed species	No	1	
		Yes	7.2 (1.7–30.2)	0.007^*^
	Agroecology	Highland	1	
		Medium	1.0 (0.3–2.9)	0.947
		Lowland	3.4 (0.8–16.2)	0.109

^*^Statistically significant.

### 3.4 Questionnaire survey analysis

#### 3.4.1 Socio-demographic characteristics of respondents

During the study period, a questionnaire survey was conducted among 100 animal owners, out of which 82 (82%) were men. The survey revealed that most respondents (47 out of 100) could not read, while 30% had completed primary education (Table 6). As per the survey, the average number of sheep and goats managed by each household was 17.9 [95% CI: 15.6–20.1, Standard Error (SE) = 1.1] and 18.6 (95 CI: 15.1–22.0, SE = 1.7).

**Table 6:** Socio-demographic characteristics of respondents in ATJK, Bora, and Ziway Dugda districts.

**Variables**	**Categories**	**Frequency**	**Percent**
Educational level	Could not read	47	47.0
	Primary	30	30.0
	Secondary	9	9.0
	Tertiary	14	14.0
Sex	Female	18	18.0
	Male	82	82.0
Age	15–30	38	38.0
	31–45	26	26.0
	>45	36	36.0
Marital status	Married	86	86.0
	Single	14	14.0
Districts	ATJK	28	28.0
	Bora	36	36.0
	Ziway Dugda	36	36.0

#### 3.4.2 The practice of respondents on the management system of sheep and goats

According to the study, 80% of the respondents sold their live animals in markets, while 20% sold them to middlemen. Additionally, 82% of the respondents reported buying live animals from livestock markets. Most of the respondents allowed their animals to drink from communal water sources and used a shared grazing system for feeding their animals (Table 7).

**Table 7:** The practice of respondents about the management system of sheep and goats.

**Variables**	**Categories**	**Frequency**	**Percent**
What is the main purpose of keeping each species?	Multipurpose	69	69.0
	Meat	31	31.0
What kind of farming system do you follow?	Sedentary mixed farming	100	100.0
How do you keep your animals at night?	Enclosed at home	100	100.0
What type of grazing system is used?	Communal	93	93.0
	Fenced	7	7.0
Where do animals drink from?	At the farm	6	6.0
	Shared water source	94	94.0
What methods did you use to sell the animals?	A direct sale to a middleman/trader	20	20.0
	Livestock market	80	80.0
What methods did you use to buy the animals	From Livestock market	82	82.0
	From middleman/trader	10	10.0
	Local neighbor or nearby village	8	8.0

#### 3.4.3 Knowledge of respondents regarding PPR in sheep and goats

According to the present study, 47% of respondents referred to PPR as “Maariyyee Hoolaa fi Re'ee” in their native language. Additionally, 46% of them were familiar with all the clinical symptoms of the disease. The study found that PPR affects both sheep and goats, as reported by 54% of the respondents. PPR has different names in various locations, depending on the symptoms it shows as depicted in Table 8.

**Table 8:** Knowledge of respondents about the PPR.

**Variables**	**Categories**	**Frequency**	**Percent**
What do you call PPR in Afan Oromo in your area?	Boossisaa	21	21.0
	Dhukkuba Sombaa	8	8.0
	Furroo	8	8.0
	Furroo fi Cittoo	6	6.0
	Maariyyee Hoolaa fi Re'ee	47	47.0
	Utaalloo	10	10.0
Do you know or have you ever experienced any of the following major symptoms of PPR?	Coughing	14	14.0
	Nasal and ocular Discharge	11	11.0
	Diarrhea	7	7.0
	Death	22	22.0
	All the above signs	46	46.0
Which animal species are affected by PPR in this area?	Sheep	19	19.00
	Goat	27	27.00
	Both	54	54.00

**Figure 2:**
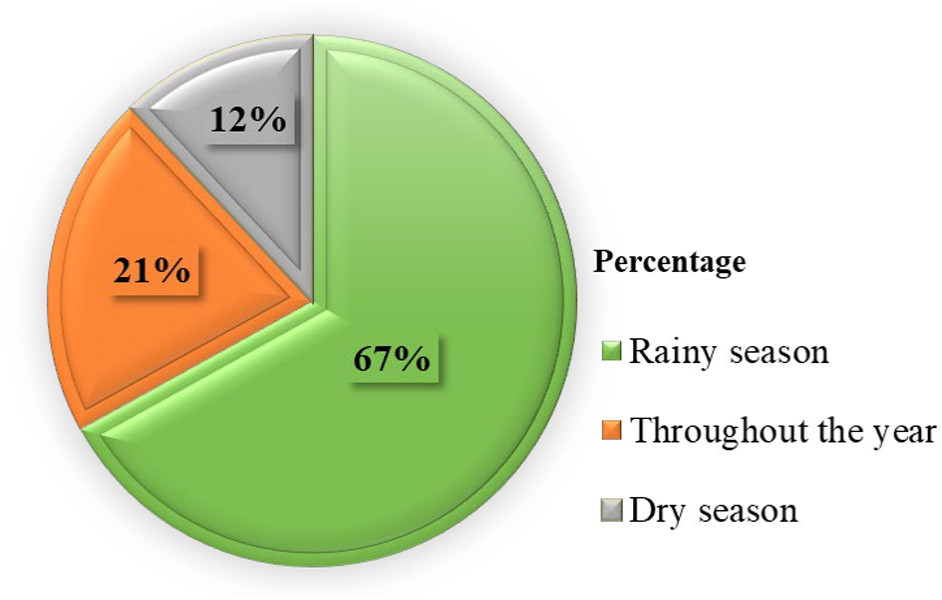
Diagram showing which season in the year PPR outbreaks happened based on the response of respondents.

Most respondents (67%) replied that the PPR outbreak happened during the rainy season in the study area (Figure 2).

#### 3.4.4 Respondents' knowledge and practices toward PPR treatment, vaccination, and outbreak

According to the present survey, 35% of respondents prefer consulting a private veterinary worker when their animals become sick. On the other hand, 28% of the respondents rely on their knowledge and treat their animals themselves. Only 37% of the respondents prefer taking their animals to a governmental veterinary service. Most respondents reported that PPR outbreaks occurred in 2017 and 2018. When asked about vaccination, 50% of the respondents didn't know whether the PPR vaccine was available in their area. Furthermore, 37% of the respondents couldn't differentiate between the PPR vaccine and regular medications they had given their animals. About 14% of the respondents said that they had vaccinated their animals 4 years ago, as presented in Table 9.

**Table 9:** Respondents' knowledge and practices toward PPR treatment, vaccination, and outbreak.

**Variables**	**Categories**	**Frequency**	**Percent**
What do you usually do if an animal is sick?	Consult a government veterinarian	37	37.00
	Consult a private veterinarian	35	35.00
	Treat it myself (or a family member)	28	28.00
Where do you get medicines to treat your animals?	General shop or market stall	16	16.00
	Government vet	42	42.00
	Private vet	42	42.00
When did you see the PPR disease outbreak recently?	2016	28	28.00
	2017	36	36.00
	2018	36	36.00
When was the PPR vaccine last given?	2014	36	36.00
	2015	14	14.00
	Not given	50	50.00

## 4 Discussion

Of the 73 flocks sampled in the present study, 71.2% (51) of them were infected with PPRV. A similar finding was reported by Taylor et al. (35) in Syria, with a 76% flock prevalence.

In the present study, 50% of sheep and goats in selected districts of the Central Oromia Region were infected with PPRV. This percentage is higher compared to the 5.71% PPR seroprevalence noted in a selected district of Horu Guduru Zone, Western Ethiopia (36). Similar PPR seroprevalence rates with current findings were seen in the Arsi and East Shewa Zones in the Oromia Region, Ethiopia (32), and the southern part of the Tigray Region, Ethiopia (37). In addition, 41.01% of PPR seroprevalence was reported in India (38), 45.8% in Tanzania (39), 55% in Nigeria (40), 55.95% in Saudi Arabia (41), and 57.6% in Uganda (42). However, the current finding was lower than that reported by Abdalla et al. (43) in Sudan, with a seroprevalence of (61.8%).

The present study showed a statistically significant difference in PPRV infection in sheep and goats in various districts. The seroprevalence of the disease in the ATJK and Bora districts was more than three times that of the Ziway Dugda district. This might be attributed to the Bora district sharing a border with the Afar Region, where there is uncontrolled movement of livestock for grazing and marketing between the pastorals of neighboring nations. Therefore, the increase in prevalence in this district could be attributed to the movement of animals that play a vital role in the transmission and maintenance of PPRV in nature (44). The present study is in line with the work done by Al-Majali et al. (45), which recorded a higher seroprevalence of PPR in regions with free animal movement than in other areas in Jordan. Moreover, the current districts have a common marketplace that could be crucial in easing contact with infected animals. Specifically, animals that were not sold and returned to the flock after the market visit could significantly contribute to the disease transmission circulation.

The current study found that sheep and goats living in lowland areas were 3.8 and 3.4 times more likely to be infected with PPR than those in highland areas. A similar finding was reported by Fournie et al. (46). Likewise, the present finding agrees with Delil et al. (47) and Megersa et al. (48) in the Afar Region, who reported that PPR prevalence was high in the lowlands. This might be explained by the fact that small ruminant flocks in lowland pastoral systems are noticeably larger and more movable in their search for grazing and watering points than in highland sedentary systems (49, 50).

The likelihood of goats contracting PPR was found to be 6.3 times higher in the current study when goat flocks had other animal species than when goats were managed separately. This could be explained by the possibility that goats could contract disease from other animals, particularly sheep.

The current study showed that one of the predictors of PPR infection in goats was the origin of the animals. The probability of PPR infection was 4.5 times greater in goats imported than those born in the flock. This can be explained by the significant risk of infection during the marketing and the potential origin of the animals might be from PPR-infected areas.

In the current research, flock size was also one of the other potential risk factors for PPR infections in sheep. The likelihood of contracting PPR also increases with flock size. For instance, the likelihood of a sheep managed in a large flock getting PPR was nine times greater than that seen in a small flock in the present study. Al-Majali et al. (45) study found that large flock size was one of the PPR risk factors.

In the present study, animal owners were aware of PPR disease, and 47% used the term “Maariyyee Hoolaa fi Re'ee” and 20% called it “Boossisaa” in the Afan Oromo language. The cited local terms are more likely to be PPR because most respondents were familiar with and had experience with the disease. For instance, 46% of the respondents were aware of PPR symptoms like coughing, diarrhea, nasal and ocular discharge with a sore mouth, and death. The reason they developed awareness about the disease might be attributed to the year-round recurrent PPR outbreaks and the regular community education programs offered by the governmental or non-governmental organizations.

In the present study, most animal owners followed poor management practices that contributed to the spread of PPR in the study area. There was a practice of keeping sheep and goats together with camels and cattle. We noticed that most species came into contact mostly at water points (94%) and during communal grazing (93%). Even though camels and cattle were not regarded as possible hosts for PPRV, El-Yuguda et al. (40) and Abraham et al. (51) reported PPRV seroconversion among these animals. Roger et al. (52) reported the first documented outbreak of PPR in camels in Ethiopia. Similarly, Lineage 4 PPRV was isolated from camels in Sudan (53). The mixing of the different species during grazing, watering, or in night enclosures (resting) between cattle and camels with small ruminants could increase the spread of PPR in the study area. Besides, all respondents put their animals at night in an enclosure with a fence from the woods, which was not built well, so that animals could escape, or wild animals could enter.

Although many people were following up on a poor management system for small ruminant production, many farmers had a positive attitude toward treating animal diseases. So, 72% of respondents were able to advise the veterinarian when they suspected PPR. Therefore, the present study result gave hope from the farmer's point of view to create applicable disease prevention and control techniques through awareness creation in the communities. Fournie (46) suggested that a change in practices among the farmers was necessary to implement disease prevention and control programs.

## 5 Limitations and future perspectives

The present study was limited to seroprevalence, assessment of potential risk factors, and community awareness toward PPR in the study area. To better comprehend PPR transmission dynamics, including its dissemination and contagiousness as well as the many roles played in this process by animal and livestock species, production methods, ecosystems, and viral lineages, a concerted epidemiological study is very crucial (54) such as investigations on disease transmission networks; modeling of small ruminant populations functioning as reservoirs; finding the genetic causes of virulence, diagnostic and surveillance techniques, molecular epidemiology, development of novel vaccines (marked and recombinant) (55).

Furthermore, to better understand the impact of PPR across all settings, a well-planned cost-benefit analysis of PPR, comparing policies and responses that incorporate both the direct and indirect impacts associated with PPR, is necessary (56).

## 6 Conclusion

The present study revealed a higher prevalence of PPR in sheep and goats in central Ethiopia, which means the disease is endemic in the area. Districts, flock size, and agroecology were independent predictors of PPRV seropositivity in sheep, while districts, origin, and mixed species were independent predictors of PPRV seropositivity in goats. Therefore, animal movement to and from the districts with caution, regular PPR vaccination, and isolation and molecular characterization of the PPR virus circulating in the study area are suggested.

## Data Availability

The raw data supporting the conclusions of this article will be made available by the authors, without undue reservation.
